# Thermal4D: Physics-Driven Gaussian Splatting for Dynamic Thermal Scene Reconstruction

**DOI:** 10.3390/s26103041

**Published:** 2026-05-12

**Authors:** Chonghao Zhong, Chao Xu

**Affiliations:** Key Laboratory of Photoelectronic Imaging Technology and System, Ministry of Education of China, School of Optics and Photonics, Beijing Institute of Technology, Beijing 100081, China; 3220230681@bit.edu.cn

**Keywords:** dynamic scene reconstruction, 3D gaussian splatting, thermal infrared imaging, frequency-aware attention, atmospheric transmission modeling

## Abstract

Dynamic scene reconstruction from thermal infrared imagery remains insufficiently studied due to several inherent challenges, including low texture, low contrast, and radiometric ambiguity. In this paper, we present Thermal4D, a novel framework for reconstructing high-fidelity dynamic 3D scenes using only thermal images, without requiring visible-light inputs or auxiliary sensors. Built upon the 3D Gaussian Splatting paradigm, the proposed method introduces two key components. First, a frequency-aware attention module, termed TherHiLo, is designed to disentangle structural features across different frequency bands. Second, a physics-inspired atmospheric transmission module (ATM) is developed to model radiometric distortions caused by thermal imaging conditions. Although the reconstruction pipeline takes 8-bit thermal video sequences as input, high-precision 14-bit thermal frames are further exploited in TherHiLo to enhance attention learning with richer radiometric information. In addition, feature-level supervision from pretrained DINOv2 models is incorporated to improve structural consistency. To facilitate systematic evaluation, we also construct MVTD, a new multi-view dynamic thermal dataset. Experimental results on the MVTD and TI-NSD benchmarks show that Thermal4D consistently outperforms existing methods in both dynamic and static scenes, providing an effective framework for physics-consistent dynamic thermal scene reconstruction.

## 1. Introduction

Dynamic 3D scene reconstruction, also referred to as 4D reconstruction, is an important task in computer vision and has broad applications in surveillance, autonomous driving, virtual reality, and complex environment perception. In recent years, visible-light-based reconstruction methods have achieved remarkable progress. However, their performance often degrades under low-light conditions, at night, or in adverse environments, which may lead to reduced robustness or even complete failure in challenging scenarios. In contrast, thermal infrared imaging provides a complementary sensing capability and can operate more reliably under such conditions, making it a promising modality for robust scene reconstruction.

**Figure 1:**
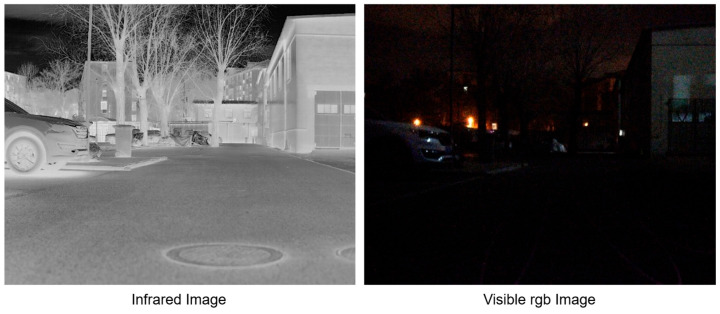
Comparison between synchronized thermal and RGB images captured from the same scene at night. The thermal image preserves clearer structural information under low-light conditions, whereas the RGB image is severely underexposed and noisy, which makes reliable feature matching and pose estimation more difficult.

All objects with a temperature above absolute zero emit infrared radiation. Thermal infrared imaging captures this radiative energy rather than reflected visible light, which makes it naturally less dependent on illumination conditions. As a result, thermal images can preserve scene structure in darkness, nighttime, and other visually degraded environments where RGB cameras often suffer from severe underexposure or noise. This characteristic makes thermal imaging particularly attractive for reconstruction tasks in challenging real-world scenarios. As shown in Figure 1, compared with a synchronized RGB image captured at night, the thermal image preserves clearer structural information under low-light conditions.

Recently, several studies have begun to explore 3D reconstruction in the thermal domain. For example, Thermal3D-GS [1] models atmospheric transmission and heat conduction using neural networks guided by thermal imaging physics, and improves static scene reconstruction through temperature consistency constraints. NTR-Gaussian [2] further introduces thermodynamic parameters, such as emissivity, convective coefficients, and heat capacity, to support more accurate thermal modeling and nighttime temperature prediction. These studies demonstrate the feasibility and potential of thermal-based 3D reconstruction. Nevertheless, existing approaches still have important limitations in practical deployment. First, most of them focus on static scenes and are not designed for dynamic environments. Second, many methods depend on auxiliary RGB inputs or external cues, such as photogrammetric point clouds or pre-estimated camera poses, which increases system complexity and limits general applicability.

Compared with visible-light images, thermal images usually exhibit lower contrast, blurrier contours, weaker texture, and stronger radiometric ambiguity. These characteristics make dynamic thermal scene reconstruction particularly challenging. In practice, directly applying visible-light-based dynamic reconstruction pipelines to thermal data often leads to degraded geometry, floaters, and unstable rendering results. A major reason is that conventional structure-from-motion pipelines [3] frequently fail to establish sufficient and reliable feature correspondences across multi-view thermal images, resulting in inaccurate or missing camera poses.

To address these issues, we propose Thermal4D, a dynamic 3D reconstruction framework that relies solely on thermal infrared video, without requiring visible-light images or auxiliary sensors. To obtain reliable initialization from thermal inputs, we adopt the Visual Geometry Grounded Transformer (VGGT) [4] to estimate camera poses and initialize sparse point clouds. Specifically, for multi-view thermal videos, we extract the first frame from each viewpoint to form a static image set, which is then processed by feed-forward VGGT to recover initial 3D attributes, including camera poses and sparse point clouds.

Built upon the 3D Gaussian Splatting (3DGS) paradigm, Thermal4D models scene dynamics through a learnable deformation field inspired by 4D Gaussian Splatting (4DGS) [5] and parameterized using K-Planes [6]. To better exploit the characteristics of thermal imagery, we further introduce TherHiLo, a hierarchical frequency-aware attention module that decouples high-frequency details from low-frequency structural information, thereby enhancing fine-grained representation while suppressing thermal noise. In addition, we allow each 3D Gaussian primitive to learn its emissivity and propose a physics-inspired atmospheric transmission module (ATM) to model radiometric distortions caused by atmospheric effects. By integrating ATM into the differentiable renderer, the proposed framework enables more physically meaningful thermal image formation and radiometric correction. Beyond combining frequency-aware thermal attention and physics-inspired rendering, the novelty of Thermal4D lies in the unified coupling of thermal feature extraction, spatio-temporal Gaussian deformation, and physics-aware thermal image formation within a thermal-only dynamic reconstruction pipeline.

**Figure 2:**
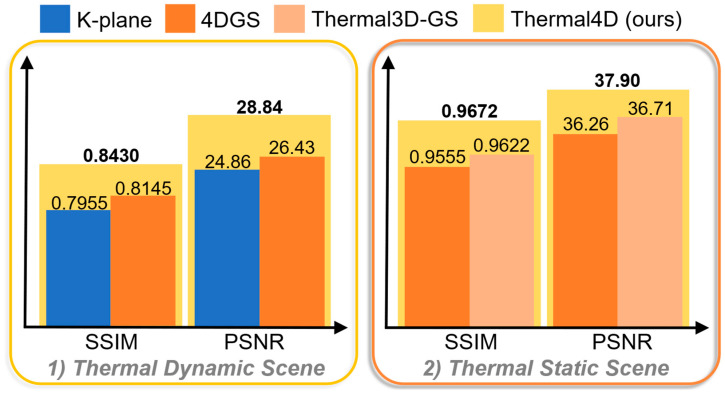
Comparison of Thermal4D with representative existing methods on dynamic and static thermal scene reconstruction tasks. The dynamic scene results are averaged over MVTD, while the static scene results are averaged over the UAV subset of TI-NSD.

To further improve reconstruction quality, we introduce feature-level supervision based on the pretrained DINOv2 model [7], which helps preserve structural consistency and reduce artifacts in rendered results. Because publicly available benchmarks for multi-view dynamic thermal reconstruction remain extremely limited, we construct MVTD as the primary benchmark for evaluating the proposed method in the dynamic setting. In addition, we include supplementary static scene evaluation to assess the generalization of the proposed framework under established thermal reconstruction settings. Specifically, we use the UAV subset of TI-NSD, which contains outdoor long-range thermal scenes captured from aerial viewpoints, to examine reconstruction performance under larger imaging distances and more challenging observation conditions. We also report results on the NTR dataset as an additional challenging thermal reconstruction setting. Figure 2 summarizes the averaged quantitative comparison on MVTD for the dynamic setting and on the UAV subset of TI-NSD for the supplementary static setting, while the NTR dataset is reported separately in the static quantitative comparison table.

The main contributions of this work are summarized as follows:

- We propose Thermal4D, a dynamic 3D reconstruction framework that operates solely on thermal infrared images, without requiring visible-light inputs or auxiliary sensors.
- We develop two key components for thermal scene modeling: TherHiLo, a frequency-aware attention module that improves thermal feature representation through high- and low-frequency decomposition, and a physics-driven atmospheric transmission module (ATM) with joint emissivity learning to better model the thermal image formation process.
- We construct MVTD, which, to the best of our knowledge, is the first benchmark for multi-view dynamic thermal scene reconstruction. The dataset is available from the corresponding author upon reasonable request, subject to institutional data-sharing policies. Extensive experiments on MVTD, together with supplementary evaluation on the UAV subset of TI-NSD and the NTR dataset, demonstrate the effectiveness of the proposed method for dynamic thermal reconstruction and its generalization to challenging static thermal scenes.

## 2. Related Work

### 2.1. Thermal 3D Reconstruction

Thermal 3D reconstruction has attracted increasing attention in recent years because of the robustness of thermal infrared imaging under low-light and adverse weather conditions. Early work has explored the feasibility of reconstructing 3D scenes directly from thermal imagery. For example, ref. [8] is among the first to introduce Neural Radiance Fields (NeRF) [9] into thermal scene reconstruction, where thermal mapping and structural thermal constraints are designed to better adapt NeRF to infrared imagery. Subsequent studies [10,11,12,13] further extend this direction by improving thermal image quality before reconstruction or by combining thermal and visible-light modalities for joint scene modeling. These methods employ temperature-aware embeddings, image translation, or voxel-based representations to alleviate the challenges of thermal images, such as low resolution, weak texture, and low contrast.

Beyond thermal-only or thermal-RGB settings, several works incorporate additional sensing modalities to improve reconstruction robustness. For instance, ref. [14] combines RGB, event, and thermal infrared cameras to improve scene reconstruction under challenging illumination conditions. Similarly, ref. [15] integrates point clouds with thermal imagery to reduce geometric ambiguity and introduces cross-modal alignment to handle spatial inconsistency across heterogeneous sensors.

More recently, thermal reconstruction methods based on 3DGS [16] have shown promising performance. Thermal3D-GS [1] incorporates neural approximations of atmospheric transmission and heat conduction into the 3DGS framework, which helps reduce floating artifacts and improve edge sharpness in static thermal scenes. NTR-Gaussian [2] further estimates thermodynamic parameters, such as emissivity, convective coefficients, and heat capacity, to support more accurate temperature reconstruction and nighttime thermal modeling. Although these methods demonstrate the effectiveness of physics-aware modeling in thermal reconstruction, most existing approaches are still mainly designed for static scenes or rely on auxiliary modalities and external priors. Dynamic 3D reconstruction from thermal imagery alone remains largely underexplored.

### 2.2. NeRF-Based Dynamic Scene Reconstruction

Dynamic scene reconstruction has been extensively studied in the NeRF literature. Existing methods can generally be grouped into several representative directions, including deformation-field-based methods, static–dynamic decomposition methods, plane-based dynamic representations, and streaming-based approaches. Deformation-field-based methods model dynamic scenes by learning a mapping from observed space–time coordinates to a canonical space. D-NeRF [17] extends NeRF with a temporal dimension and learns a spatio-temporal radiance field for dynamic scenes. Nerfies [18] focuses on non-rigid scenes and introduces deformation modeling for human-centered dynamic content. HyperNeRF [19] further addresses topological changes by extending the representation into a higher-dimensional space. Another line of work separates static and dynamic components to improve learning efficiency and scene disentanglement. For example, ref. [20] decomposes video content into static and dynamic parts in a self-supervised manner, while NeRFPlayer [21] improves the flexibility of dynamic NeRFs by allowing the appearance of newly introduced objects. To improve efficiency, plane-based dynamic representations have also been widely adopted. HexPlane [22] decomposes 4D space into multiple plane combinations for efficient feature encoding, while K-Planes [6] generalizes this idea by projecting coordinate pairs onto learnable feature planes. In addition, streaming-based methods such as OD-NeRF [23] aim to support progressive or online reconstruction without requiring access to the complete sequence in advance. These methods have significantly advanced dynamic scene modeling. However, most of them are developed for visible-light imagery and do not explicitly consider the sensing characteristics of thermal infrared images, such as weak texture, low contrast, and radiometric distortions.

### 2.3. 3DGS-Based Dynamic Scene Reconstruction

Recent advances in 3DGS [16] have extended Gaussian-based representations from static scenes to dynamic scene reconstruction. Compared with NeRF-based methods, dynamic 3DGS approaches often provide improved rendering efficiency while maintaining high visual fidelity, which has led to growing interest in temporal Gaussian modeling.

One important direction is to model dynamic Gaussians explicitly over time. Several methods represent Gaussian motion using continuous trajectories, sparse keyframes, or deformation functions [5,24,25,26,27]. These approaches improve temporal smoothness and reduce discretization of artifacts in dynamic rendering. Other studies further decompose motion into more interpretable components or track persistent Gaussians over time, enabling compact and controllable dynamic representations [28,29,30].

Another research direction introduces motion priors or physical constraints into dynamic Gaussian modeling. For example, refs. [31,32,33] incorporate optical flow or velocity priors to better disentangle scene motion and camera motion. Some methods further integrate physical simulation or material properties into the reconstruction process [31,34,35], enabling more physically plausible modeling of deformable or fluid scenes.

To improve scalability in long or streaming video scenarios, several methods propose memory-efficient and online learning strategies [36,37,38]. Other works focus on robust deformation learning, sparse-view reconstruction, hybrid representations, and challenging real-world capture settings [39,40,41,42,43,44,45,46,47]. More recently, 4DGT [48] learns a feed-forward Transformer to directly predict consistent 4D Gaussians from monocular videos, improving the efficiency and scalability of dynamic scene reconstruction. In addition, recent studies on attention-guided feature enhancement in other vision tasks [49,50] suggest that multi-scale feature interaction can improve representation robustness in challenging imaging conditions, which is also relevant to the design of our thermal feature modeling.

Although dynamic 3DGS methods have achieved rapid progress, they are primarily designed for visible-light imagery and general dynamic scenes. They usually do not explicitly account for the unique properties of thermal infrared imaging, such as low texture, low contrast, and atmosphere-related radiometric degradation. This limits their direct applicability to thermal dynamic scene reconstruction and motivates the development of a dedicated thermal dynamic 3DGS framework.

## 3. Preliminaries: 3D Gaussian Splatting

3DGS represents a scene as a set of anisotropic Gaussian primitives in 3D space. Each Gaussian is parameterized by its center position, scale, rotation, opacity, and color, where the color is typically represented using spherical harmonics. Owing to its explicit and differentiable formulation, 3DGS provides an efficient representation for high-quality novel view synthesis.

Formally, each Gaussian primitive is defined by a mean $\;\mu \in \;R^{3}\;$ and a covariance matrix $\Sigma \in R^{3\;\times \;3}$. The Gaussian function can be written as

(1) $$G(x)=\exp\left(-\frac{1}{2}{\left(x-\mu \right)}^{T}\Sigma^{-1}(x-\mu )\right)$$

To ensure that Σ is positive semi-definite, it is parameterized as

(2) $$\Sigma =RSS^{T}R^{T}$$

where S is a diagonal scaling matrix and R is a rotation matrix.

During rendering, each 3D Gaussian is projected onto the image plane using the camera transformation and a local affine approximation of the perspective projection. The corresponding 2D covariance is written as

(3) $$\Sigma^{\prime }=JW\Sigma W^{T}J^{T}$$

where W denotes the camera transformation matrix and J is the Jacobian of the projection function. The rendered pixel color is obtained by front-to-back alpha compositing over the Gaussians intersecting the viewing ray:

(4) $$C(x)=\sum_{i\in N}c_{i}f_{i}(x)\prod_{j=1}^{i-1}\left(1-f_{j}(x)\right)$$

where N denotes the set of contributing Gaussians, $c_{i}$ is the color of the *i*-th Gaussian, and $f_{i}\left(x\right)$ is its opacity contribution at pixel location x, defined as

(5) $$f_{i}(x)=\alpha_{i}\exp\left(-\frac{1}{2}{\left(x-\mu_{i}\right)}^{T}\Sigma_{i}^{-1}(x-\mu_{i})\right)$$

where $\alpha_{i}\;$ denotes the opacity of the *i*-th Gaussian.

In this work, we build upon the 3DGS framework and extend it to dynamic thermal scene reconstruction by introducing temporal deformation modeling, frequency-aware thermal feature learning, and physics-inspired thermal image formation.

## 4. Method

**Figure 3:**
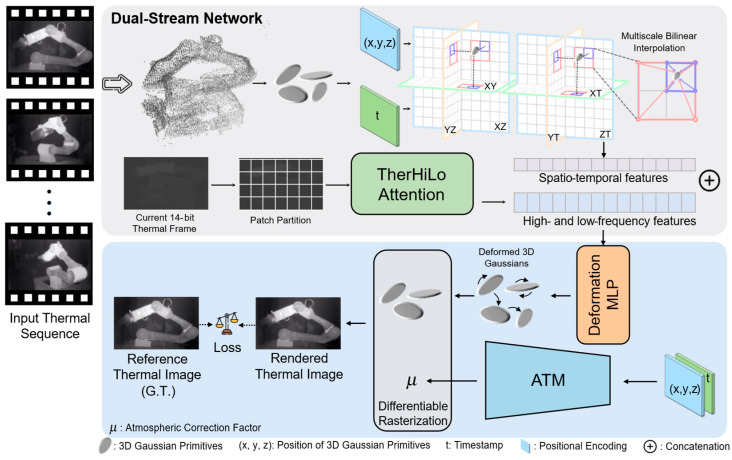
Overview of the Thermal4D pipeline. Multi-view 8-bit thermal video is used to estimate camera poses and initialize a static reconstruction via VGGT and 3DGS. Temporal dynamics are modeled by K-Plane encoding, while the current frame’s 14-bit image is processed by the TherHiLo module to extract frequency-aware features. These are fused through an MLP to predict Gaussian deformations over time. An atmospheric transmission module (ATM) imposes physical constraints to improve rendering fidelity.

Figure 3 illustrates the overall pipeline of the proposed Thermal4D framework. Given a synchronized multi-view thermal video sequence in 8-bit format, we first apply VGGT to the first frame from each view to estimate camera poses and recover an initial sparse point cloud. These outputs are then used to initialize a static scene representation based on 3DGS, forming the coarse stage of the reconstruction pipeline.

To model temporal dynamics, we introduce a spatio-temporal encoder that maps each Gaussian primitive and its timestamp into a compact motion feature. In parallel, the corresponding 14-bit thermal frame at the current time step is processed by the proposed TherHiLo module to extract frequency-aware image features that preserve subtle thermal variations. The spatio-temporal features and thermal attention features are then fused and passed through an MLP to predict Gaussian deformations, enabling the generation of frame-specific Gaussian primitives in the dynamic stage.

To further improve physical consistency, we introduce an Atmospheric Transmission Module (ATM), which models radiometric attenuation and path radiation effects in thermal image formation. The entire framework is optimized using a combination of reconstruction, perceptual, feature-level, and regularization losses, including DINOv2-guided supervision and temporal smoothness constraints for dynamic scenes.

### 4.1. Deformation Field-Based 4D Gaussian Splatting

Following deformation-based dynamic 3DGS methods, we model a dynamic scene as a temporal deformation of a canonical static scene. In the standard static 3DGS formulation, each Gaussian primitive is represented by its position, rotation, scale, opacity, and spherical harmonic coefficients. In the dynamic setting, an additional timestamp t is introduced so that the Gaussian attributes can vary over time.

Let G={x,y,z}  denote the 3D position of a Gaussian primitive at the canonical state. To capture temporal motion, we employ a spatio-temporal encoder H, which takes the primitive position and timestamp as input and outputs a low-dimensional motion feature:

(6) f=H(G,t)

Specifically, the 4D coordinate (x,y,z,t) is represented using K-Plane encoding, where the spatio-temporal space is factorized into six 2D planes, namely xy, xz, yz, xt, yt, and zt. Multi-scale bilinear interpolation is then applied on these feature planes to obtain the encoded spatio-temporal feature f.

Given the encoded feature f, a deformation decoder D, implemented as a multi-head MLP, predicts the deformation of the Gaussian primitive:

(7) ΔG=D(f)={Δx,Δy,Δz,Δr,Δs}

where Δx,Δy,Δz denote positional offsets, and Δr and Δs represent changes in rotation and scale, respectively. The deformed Gaussian primitive at time step t is then given by

(8) $$G^{\prime }=G+\Delta G$$

Finally, the deformed Gaussians are rendered using the differentiable rasterization process of 3DGS:

(9) $$\hat{I}=S(M,G^{\prime })$$

where $\hat{I}$ is the rendered image and M denotes the camera parameters. This formulation allows the model to represent scene motion as continuous Gaussian deformation over time.

### 4.2. Thermal High–Low Attention (TherHiLo)

#### 4.2.1. Attention Module

Thermal infrared images typically contain limited high-frequency texture and dominant low-frequency structures. However, the weak high-frequency components, such as object boundaries and subtle edge transitions, often provide important cues for motion estimation and deformation prediction. At the same time, thermal images are also prone to noise-induced pseudo-motion, especially in frequency-sensitive representations. This makes it necessary to explicitly distinguish informative structural details from noise.

To address this issue, we propose a frequency-aware attention module, termed TherHiLo, which decomposes thermal representations into high- and low-frequency components and processes them using two parallel branches. The high-frequency branch focuses on local edges and contour information that are beneficial for deformation estimation, while the low-frequency branch captures global thermal distributions and structural context. By combining the two, TherHiLo improves the robustness of feature extraction and helps suppress spurious motion patterns caused by thermal noise.

TherHiLo is built upon the HiLo attention architecture and includes three key modifications: a learnable gating parameter α, a shifted window attention mechanism, and a blur-pooling strategy. Unlike the original HiLo design, which uses a fixed hyperparameter to allocate attention heads, our method introduces a learnable scalar α to adaptively determine the balance between high- and low-frequency attention according to the input content.

For each attention block, the numbers of high- and low-frequency heads are defined as

(10) $$N_{h}^{Hi}=\lfloor (1-\sigma (\alpha ))\cdot N_{h}\rfloor \;N_{h}^{Lo}=N_{h}-N_{h}^{Hi}$$

where $N_{h}$ is the total number of attention heads and $\sigma \left(\cdot \right)$ denotes the sigmoid function. This design enables the network to dynamically adjust its frequency emphasis for different scenes and image regions.

To improve information exchange across local windows, the high-frequency branch adopts shifted window attention. Specifically, consecutive high-frequency attention layers alternate between regular window partitioning and shifted window partitioning. This strategy expands the effective receptive field while preserving the computational efficiency of local attention, allowing the network to better capture continuous edge structures across neighboring windows. The shift size is set to half of the window size, i.e., $\Delta =\left\lfloor ws/2\right\rfloor$, where ws denotes the window size.

In the low-frequency branch, we replace conventional average pooling with blur pooling to reduce structural degradation during downsampling. Specifically, a Gaussian kernel is applied before subsampling:

(11) $$x_{lo}=\mathrm{Downsample}(G_{\sigma }*x)$$

where ∗ denotes convolution, $G_{\sigma }$ is a 3×3 Gaussian kernel. In our implementation, σ is fixed to 1.0 and is not learnable. Compared with average pooling, which performs uniform local averaging and may introduce stronger aliasing and boundary distortion during subsampling, blur pooling first applies low-pass filtering before downsampling. This operation better respects the sampling principle by suppressing high-frequency noise that cannot be reliably preserved at lower resolution, while retaining the dominant low-frequency structural pattern. As a result, blur pooling reduces aliasing artifacts and preserves structural coherence more effectively, which is beneficial for modeling large-scale thermal distributions in the low-frequency branch.

#### 4.2.2. Why 14-Bit Thermal Input Is Used in TherHiLo

In many long-wave infrared imaging systems, the raw 14-bit sensor data are converted into 8-bit images through dynamic range compression operations such as automatic gain control or histogram equalization. Although these operations improve visual contrast for human observation, they inevitably discard part of the original radiometric information. As a result, the 8-bit representation often loses subtle thermal variations and weak structural cues that are important for feature extraction and motion modeling.

To alleviate this issue, TherHiLo directly operates on the original 14-bit thermal data rather than the AGC-compressed 8-bit images. Because the 14-bit input preserves the full sensor dynamic range and finer radiometric detail, it provides richer information for frequency-aware feature learning. This is particularly useful in regions with smooth thermal transitions or weak boundaries, where compression may obscure meaningful structures.

**Figure 4:**
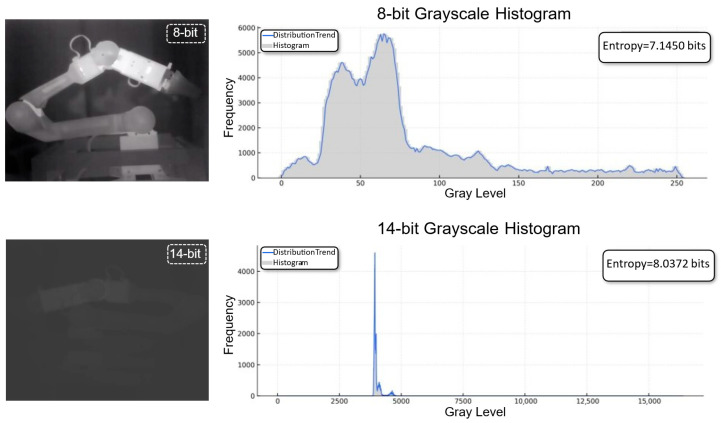
Comparison between 8-bit AGC-compressed and raw 14-bit thermal images. The 14-bit representation preserves a wider dynamic range and richer radiometric details, which are beneficial for structural perception and thermal feature extraction.

As shown in Figure 4, the 14-bit representation retains richer intensity variation and more complete radiometric information than the corresponding 8-bit image. This motivates the use of 14-bit thermal frames in the TherHiLo branch, while the overall reconstruction pipeline still operates on the standard 8-bit thermal video sequence.

#### 4.2.3. Network Architecture of TherHiLo

**Figure 5:**
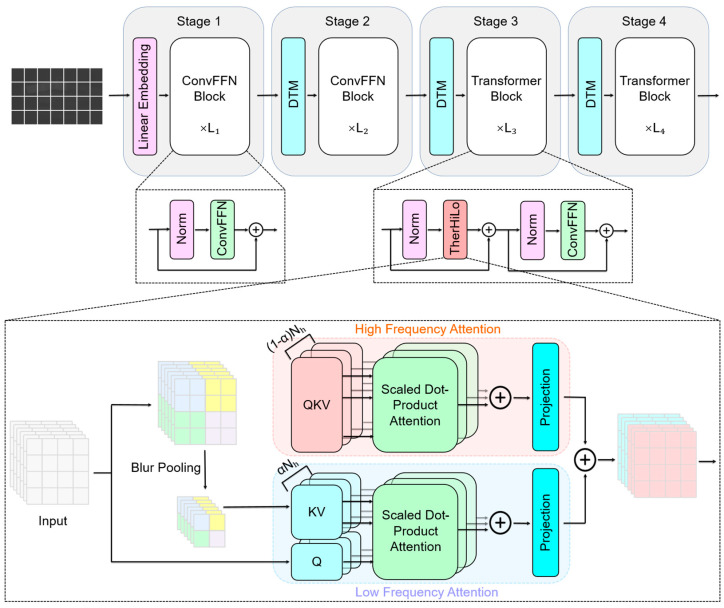
Overall architecture of the proposed TherHiLo module. The figure shows the four-stage hierarchical network together with the internal design of the TherHiLo block, including the high-frequency branch, the low-frequency branch, blur pooling, and adaptive feature fusion.

The overall architecture of the proposed TherHiLo module is shown in Figure 5. It adopts a four-stage hierarchical design to progressively extract and condense feature representations from the input thermal image patches.

The input thermal image is first divided into non-overlapping patches and is linearly projected into token embeddings. These tokens are then processed through four stages of hierarchical representation learning.

Stage 1 consists of a linear embedding layer followed by $L_{1}$ stacked ConvFFN blocks. Each block includes normalization, a convolutional feed-forward network, and a residual connection. This stage preserves the original token resolution while enhancing local feature representation. Stage 2 reduces the spatial resolution through a Deformable Token Merging (DTM) module, which performs learnable and spatially adaptive downsampling. The resulting tokens are then processed by $L_{2}$ ConvFFN blocks. Stage 3 further downsamples the representation and increases the feature dimensionality. It contains $L_{3}$ Transformer blocks, each composed of two submodules: a normalization layer followed by TherHiLo attention and residual connection, and another normalization layer followed by a ConvFFN and residual connection. Stage 4 follows the same design as Stage 3, with an additional DTM module and $L_{4}$ Transformer blocks to refine global contextual information at the coarsest scale.

**Figure 6:**
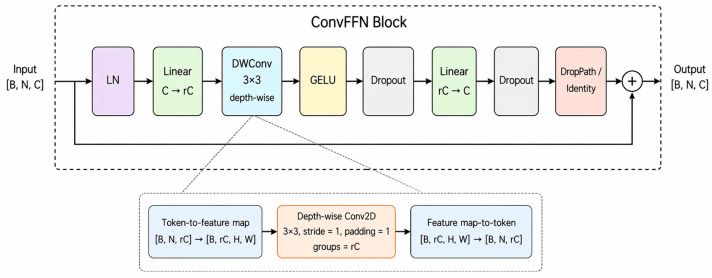
Structure of the ConvFFN block used in the early stages of TherHiLo. The block consists of LayerNorm, channel expansion, depth-wise convolution, GELU, dropout, linear projection, and a residual connection with DropPath. The inset illustrates the token-to-feature-map and feature-map-to-token operations used by the depth-wise convolution.

In our implementation, the numbers of blocks in the four stages are set to $L_{1}=2,{\;L}_{2}=2,{\;L}_{3}=4,{\;L}_{4}=2$, respectively. As shown in Figure 6, the ConvFFN block used in the first two stages is adapted from the ConvFFN design in HiLo attention [50] follows a pre-normalization residual design. It first applies LayerNorm to the input tokens, expands the channel dimension through a linear layer, and then performs depth-wise 3 × 3 convolution to introduce local spatial interaction. After GELU activation and dropout regularization, the features are projected back to the original channel dimension and added to the shortcut branch through DropPath. This design enhances local representation learning while maintaining low computational overhead in the early stages of TherHiLo. Overall, the combination of ConvFFN, DTM, and TherHiLo attention enables the network to capture both fine local thermal details and large-scale structural context in a frequency-aware manner.

#### 4.2.4. Feature Query and Fusion

The spatio-temporal feature produced by the K-Plane encoder is not directly used for Gaussian deformation prediction. Instead, it is fused with frequency-aware image features extracted by TherHiLo from the corresponding 14-bit thermal frame. In this way, the deformation field is conditioned not only on the spatio-temporal coordinates of each Gaussian primitive but also on image evidence from the current view.

Specifically, given a Gaussian primitive located at position (x,y,z)  and timestamp t, the K-Plane module first produces a spatio-temporal feature vector $F_{kp}$. Meanwhile, the corresponding 14-bit thermal image is processed by TherHiLo to generate a dense attention feature map $F_{HiLo}$. Each 3D Gaussian center is then projected onto the image plane using the camera intrinsics and extrinsics, and its corresponding feature is sampled from the TherHiLo feature map by bilinear interpolation. The sampled image feature is concatenated with the K-Plane feature to form the fused representation $F_{fused}=[F_{kp};F_{HiLo}]$, which is then fed into the deformation MLP to regress Gaussian deformation parameters. This design enables the deformation prediction to jointly leverage spatio-temporal motion cues and frequency-aware thermal appearance cues, rather than using thermal features only as an auxiliary image representation, thereby distinguishing Thermal4D from a straightforward combination of attention enhancement and physics-based rendering. The overall procedure is summarized in Algorithm 1.
**Algorithm 1. View-guided Feature Querying via Attention Map Sampling****Input:** 3D Gaussian centers, camera intrinsics and extrinsics, input thermal image, TherHiLo feature extractor, and K-Plane features.**Output:** Fused features for deformation prediction.**Step 1.** Resize the input thermal image to a fixed resolution and extract the TherHiLo feature map.**Step 2.** Project the 3D Gaussian centers onto the image plane using the camera intrinsics and extrinsics.**Step 3.** Normalize the projected coordinates to the feature-map coordinate system.**Step 4.** Apply a visibility mask and sample valid TherHiLo features from the feature map using bilinear interpolation.**Step 5.** Concatenate the sampled TherHiLo features with the K-Plane features to obtain the fused representation for deformation prediction.

### 4.3. Atmospheric Transmission Module

To account for radiative attenuation and background radiation in long-wave infrared imaging, we introduce an Atmospheric Transmission Module (ATM) to model the propagation of emitted thermal radiation from each Gaussian primitive. In our formulation, the color of each Gaussian primitive corresponds to an approximate surface temperature. The ATM then corrects this temperature-related radiative signal according to physically meaningful atmospheric factors.

Specifically, for each Gaussian primitive, the module predicts two scalar quantities: atmospheric transmittance and atmospheric path radiance. These are denoted by $\mu_{\tau }$ and $\mu_{L_{a}}$, respectively. The first term models the attenuation of object-emitted radiation during transmission, while the second term represents the thermal radiation accumulated from the atmosphere along the optical path.

Each Gaussian primitive is also assigned a learnable emissivity ϵ, which is optimized during the coarse static stage and fixed during the dynamic stage. Given the spatial position $P\in R^{3}$ and timestamp t∈R, a lightweight MLP predicts $\mu_{\tau }\;$ and $\mu_{L_{atm}}$ for each primitive.

The spherical harmonic coefficients of each Gaussian are first converted into an approximate surface temperature T. This temperature is then transformed into radiative intensity using Planck’s law:

(12) $$B_{\lambda }(\lambda ,T)=\frac{2hc^{2}}{\lambda^{5}}\cdot \frac{1}{e^{\frac{hc}{\lambda kT}}-1}$$

where h is Planck’s constant, c is the speed of light, k is Boltzmann’s constant, and λ is the wavelength. In our implementation, λ is set to 10.5 µm according to the average spectral response of the sensor.

The corrected radiance of each Gaussian primitive is then computed as

(13) $$\mu_{Ltotal}=\mu_{T}\cdot \epsilon \cdot B(T)+(1-\mu_{T})\cdot \mu_{Latm}$$

The first term corresponds to the object-emitted radiance after atmospheric attenuation, and the second term models the path radiance introduced by the atmosphere. The corrected radiance $\mu_{L_{total}}$ is finally used as the input color in the Gaussian splatting renderer, thereby improving the physical consistency of thermal image synthesis.

### 4.4. Loss Function

The proposed method is trained by jointly optimizing photometric, perceptual, feature-level, and regularization objectives. The basic reconstruction term is defined as the pixel-wise $L_{1}$ loss between the rendered image and the ground-truth image, which enforces accurate image reconstruction at the pixel level.

To improve structural and perceptual quality, we further incorporate the SSIM loss and LPIPS loss. These two terms encourage the rendered output to preserve local structure and perceptual similarity beyond simple pixel matching.

To introduce higher-level structural guidance, we employ a feature supervision term based on DINOv2. Specifically, deep features are extracted from both the rendered image and the ground-truth image, and an $L_{2}$ distance is computed between them. Although DINOv2 is pretrained on natural images, we use it here only as an auxiliary feature-level regularizer rather than as a thermal-domain semantic backbone. The motivation is that its intermediate representations can still provide useful structural cues, such as object layout, region boundaries, and contour continuity, which help regularize the consistency between rendered and target thermal images despite the modality gap. As also supported by the ablation results, this feature-level supervision improves structural consistency and perceptual quality in the reconstructed thermal scenes.

For dynamic scene reconstruction, we additionally apply a total variation regularization term during the dynamic training stage. Following the regularization strategy used in K-Plane-based representations, this term promotes smooth variation across the learned feature planes and helps stabilize temporal deformation.

The total loss is defined as

(14) $$L_{total}=L_{recon}+\lambda_{SSIM}L_{SSIM}+\lambda_{LPIPS}L_{LPIPS}+\lambda_{DINO}L_{feat}+\lambda_{TV}L_{TV}$$

where $\lambda_{ssim}=0.1$, $\lambda_{LPIPS}=0.1$, $\lambda_{DINO}=0.001$, and $\lambda_{TV}=0.1\;$ in our implementation. The total variation term is only applied during the dynamic fine-training stage.

## 5. Experiments

### 5.1. Experimental Settings

#### 5.1.1. Baseline Methods

We compare the proposed method with representative existing approaches for both dynamic and static thermal scene reconstruction. Although our primary focus is dynamic thermal reconstruction, we also evaluate static scenes to further examine the generalization ability and reconstruction fidelity of Thermal4D. A static scene can be regarded as a special case of the dynamic reconstruction setting in which temporal variation is absent. Moreover, because publicly available benchmarks for multi-view dynamic thermal reconstruction remain extremely limited, supplementary evaluation on static thermal datasets provides additional evidence regarding geometric accuracy, radiometric consistency, and novel view synthesis quality.

For dynamic scenes, we consider several representative baselines, including K-Planes [6], ENeRFi [51], Instant-NGP+T [51], and 4DGS [5]. These methods are selected because they explicitly support temporal scene modeling and can be adapted to the multi-view dynamic thermal reconstruction setting considered in this work. K-Planes models 4D spatio-temporal radiance fields through planar factorization with an efficient linear decoder. ENeRFi and Instant-NGP+T are implemented within the EasyVolcap framework [49] and adapted to thermal video inputs for dynamic novel view synthesis. 4DGS extends 3DGS to dynamic scenes by introducing time-dependent Gaussian deformation and supports efficient rendering with high visual fidelity. For fairness, all dynamic baselines are evaluated under the same image resolution, camera settings, and multi-view configuration.

For supplementary static scene evaluation, we compare against 3DGS [16], Thermal3D-GS [1], NTR-Gaussian [2], and 4DGS [5]. 3DGS serves as a generic Gaussian-based static reconstruction baseline. Thermal3D-GS is specifically designed for thermal scene reconstruction and incorporates atmospheric transmission and thermal conduction modeling. NTR-Gaussian further introduces physically meaningful thermodynamic parameters, such as emissivity and related thermal properties, to improve nighttime thermal reconstruction and temperature estimation across different time points. Thermal3D-GS is included because it is a representative thermal-specific static baseline, while NTR-Gaussian is included because it is a strong thermal-specific method for temporal thermal modeling. However, neither method is originally formulated for multi-view dynamic thermal scene reconstruction from thermal video with explicit temporal deformation. To the best of our knowledge, there are currently no established thermal-specific baselines that explicitly support multi-view dynamic thermal scene reconstruction in the same setting as MVTD. We also include 4DGS in the static setting to examine whether a dynamic Gaussian framework can generalize to static thermal scenes. All methods are evaluated using the same camera intrinsics, image resolution, and view configuration whenever applicable.

#### 5.1.2. Dataset

To evaluate the proposed method in the dynamic thermal reconstruction setting, we construct a new dataset named MVTD, which contains several real-world dynamic scenes captured exclusively in the thermal domain. All data are recorded using a FLIR Tau2 640 long-wave infrared camera with a spatial resolution of 640 × 512 pixels and a spectral response range of 7.5–13.5 µm.

The MVTD dataset is designed to cover diverse motion patterns and thermal characteristics. It contains three representative scenarios. The first is an oscillating thermal source, in which an infrared halogen heater (“Little Sun”) rotates continuously and produces a localized, highly dynamic thermal signal. The second is an articulated robotic arm, which exhibits non-rigid motion, self-occlusion, and complex structural deformation. The third is a rotating heating platform, in which a temperature-controlled surface fixed at 55 °C is mounted on a motorized turntable, generating globally consistent motion with stable thermal emission.

All sequences are captured using a single thermal camera under a software-synchronized acquisition protocol. Specifically, for each viewpoint, thermal video recording is started simultaneously with the onset of scene motion, and the same repeatable dynamic process is performed across different viewpoints to obtain multi-view observations. For each scene, we collect thermal videos from approximately 20 viewpoints. After preprocessing and data-quality screening, 17 viewpoints are retained for the final evaluation in the Robotic Arm and Heating Platform sequences, while all 20 viewpoints are retained in the Little Sun sequence. Accordingly, Table 1 reports the number of successfully registered views over the number of evaluated views for each sequence. To support both standard reconstruction and radiometrically informed modeling, we record both 8-bit AGC-processed thermal videos and the corresponding raw 14-bit thermal videos for each viewpoint.

For supplementary static scene evaluation, we use the UAV subset of the publicly available TI-NSD dataset, which contains outdoor thermal scenes captured from aerial viewpoints. We specifically select this subset because it provides long-range thermal observations that are complementary to MVTD, whose scenes are collected indoors. This allows us to further assess the generalization of the proposed method under larger imaging distances and more challenging outdoor thermal conditions. In addition, we include the NTR dataset to further compare with NTR-Gaussian under a challenging thermal reconstruction setting. Together, the UAV subset of TI-NSD and the NTR dataset serve as supplementary static benchmarks, complementing MVTD for evaluating reconstruction fidelity, geometric consistency, and physics-aware thermal rendering. Although MVTD serves as the primary benchmark for dynamic thermal reconstruction, it currently contains three indoor scenarios covering distinct motion and thermal characteristics.

#### 5.1.3. Evaluation Metrics

We use three widely adopted metrics to quantitatively evaluate novel view synthesis quality: PSNR, SSIM, and LPIPS. PSNR measures pixel-level reconstruction fidelity between the rendered image and the ground-truth image. A higher PSNR indicates lower photometric error. SSIM evaluates structural similarity by comparing luminance, contrast, and local structure, and is particularly useful for assessing structural consistency in thermal imagery. LPIPS measures perceptual similarity in a deep feature space using pretrained image representations, where lower values indicate better perceptual agreement. Together, these three metrics provide complementary evaluation from the perspectives of pixel accuracy, structural preservation, and perceptual quality.

#### 5.1.4. Implementation Details

All experiments are conducted in PyTorch 1.13.1 on a single NVIDIA RTX 3090 GPU. For dynamic scenes, Gaussian primitives are initialized using sparse point clouds and camera poses estimated by VGGT. Specifically, the synchronized multi-view 8-bit thermal videos are first decomposed into image frames, and the first frame from each view is used for pose estimation and static initialization. The confidence threshold in VGGT is set to 80%. The corresponding 14-bit thermal videos are also decomposed into frames and aligned temporally with the 8-bit sequences.

During training, each 14-bit thermal frame is partitioned into non-overlapping patches and processed by the TherHiLo module. TherHiLo is used in both the coarse and fine stages. Each Gaussian primitive is assigned an emissivity value initialized to 0.95. The emissivity is optimized only during the coarse stage and then fixed during the dynamic fine stage. We jointly optimize Gaussian positions, scales, rotations, spherical harmonic coefficients, opacities, and emissivities using the Adam optimizer with an initial learning rate of $1\times 10^{-2}$ and a cosine annealing schedule. A batch of four views is used during training, and the full model, including TherHiLo and the atmospheric transmission module, is optimized end-to-end for 30k iterations.

For the dynamic baselines, all methods are trained from scratch using the same image resolution (640×512), camera poses, and multi-view setting. For K-Planes, we use a unified 4D configuration with input coordinate dimension 4, grid dimension 2, resolution [64, 64, 64, 200], multiresolution factors [1, 2], and a total of 30 k iterations (3 k coarse and 27 k fine), with the learning rate decayed from $8\times 10^{-4}$ to $8\times 10^{-7}$. ENeRFi and Instant-NGP+T are evaluated using the EasyVolcap implementation with thermal inputs under the same training views and resolution. For static scene evaluation, 3DGS follows the official implementation, while Thermal3D-GS and NTR-Gaussian are evaluated according to their respective public settings as closely as possible under our data preprocessing pipeline.

#### 5.1.5. Evaluation Protocol

For both dynamic and static experiments, we adopt an LLFF-style holdout protocol with llffhold = 8. Specifically, one out of every eight images is held out for testing, while the remaining images are used for training. For the dynamic sequences in MVTD, all processed frames are used in the evaluation, and the reported PSNR, SSIM, and LPIPS values are averaged over all held-out test images of each sequence. Unless otherwise stated, all quantitative results are reported from a single run after stable training.

### 5.2. Visual Analysis of the TherHiLo Attention Mechanism

To better understand the behavior of the proposed frequency-aware attention module, we visualize the learned high-frequency and low-frequency components of TherHiLo using a real thermal image from the robotic arm sequence. The original image has a resolution of 640×512 pixels and is divided into non-overlapping 16×16 patches, following the input setting used in TherHiLo. We extract the attention maps after the first TherHiLo block for visualization and analysis. 

#### 5.2.1. Attention Map Visualization

**Figure 7:**
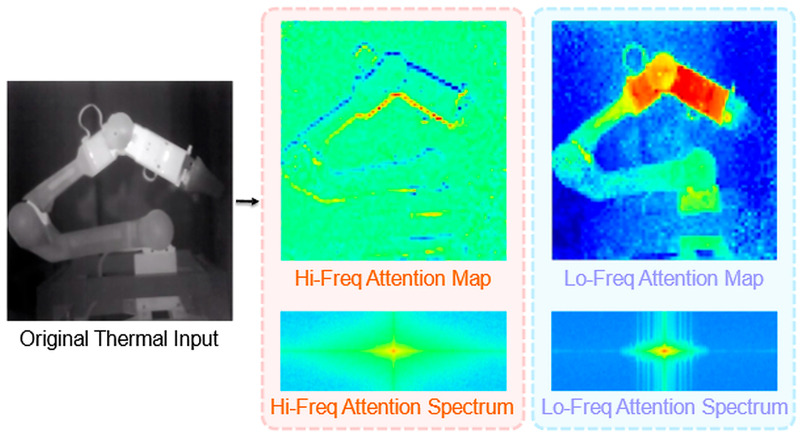
Visual and spectral analysis of TherHiLo attention. (**Left**): input 14-bit thermal image. (**Middle**): high-frequency attention map and its spectrum, highlighting structural transitions such as joints and boundaries. (**Right**): low-frequency attention map and its spectrum, emphasizing smooth global thermal patterns. The results illustrate effective frequency separation in TherHiLo.

As shown in Figure 7, TherHiLo separates the learned attention responses into two complementary components. The low-frequency branch focuses on global thermal structure, such as the overall contour of the robotic arm and smoothly varying temperature regions. Its activation is spatially coherent and concentrates on large-scale regions dominated by low-frequency information.

In contrast, the high-frequency branch emphasizes localized variations, particularly around joints, edges, and structural boundaries. Although the attention maps are computed on patch-based features, the high-frequency component still clearly highlights the discontinuities that are important for fine-grained geometric reasoning and deformation estimation.

#### 5.2.2. Frequency-Domain Analysis

To further examine the frequency selectivity of the learned attention maps, we compute the 2D Fourier transform of the reconstructed attention grids and visualize the log-magnitude spectra with frequency centering. As expected, the low-frequency branch exhibits energy concentrated near the spectral center, indicating sensitivity to smooth global patterns. By contrast, the high-frequency branch shows a broader spectral distribution, reflecting stronger responses to localized structural variation.

These results confirm that TherHiLo effectively separates thermal information into complementary frequency components. This property is particularly beneficial for thermal dynamic reconstruction, where low contrast, weak texture, and sensor noise make feature extraction more challenging than in visible-light imagery.

### 5.3. Qualitative Comparisons

We qualitatively compare Thermal4D with representative baselines on both dynamic and static thermal scene reconstruction tasks.

**Figure 8:**
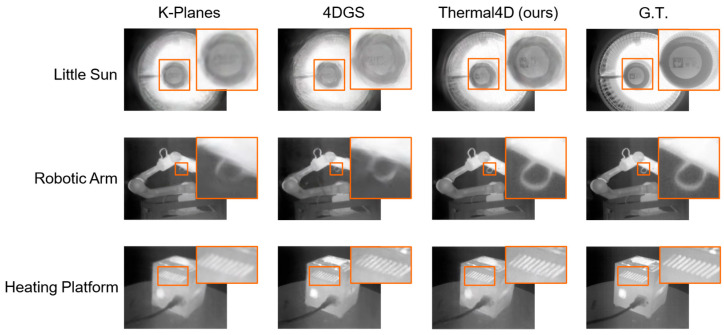
Qualitative comparison on dynamic thermal scenes from the MVTD dataset. Zoomed-in regions highlight differences in moving structures, thermal boundaries, and local reconstruction fidelity.

For dynamic scenes, Figure 8 presents the results on three representative MVTD sequences: Little Sun, Robotic Arm, and Heating Platform. Each row corresponds to one scene, and each column shows the output of a different method. Zoomed-in regions are included to highlight local differences in moving structures, thermal boundaries, and detail preservation. Compared with K-Planes, ENeRFi, Instant-NGP+T, and 4DGS, the proposed Thermal4D reconstructs more complete geometry and produces sharper thermal boundaries, especially around articulated motion, localized heat sources, and regions with thermal discontinuity. The competing methods tend to suffer from blurry boundaries, incomplete structures, or unstable appearance in regions with complex motion and weak texture.

**Figure 9:**
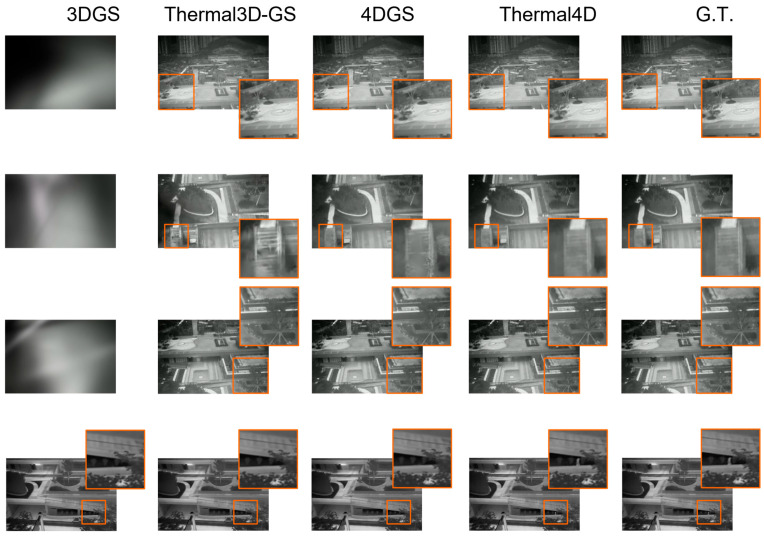
Qualitative comparison on a static UAV-captured scene from the TI-NSD dataset. The proposed method preserves geometric structure and fine thermal details more faithfully than the competing methods.

For static scenes, Figure 9 shows qualitative results on the TI-NSD dataset. Our method preserves finer geometric structures and clearer thermal contours, while maintaining better spatial continuity in low-contrast regions. In comparison, 3DGS, Thermal3D-GS, NTR-Gaussian, and 4DGS all exhibit varying degrees of detail loss, boundary blur, or structural incompleteness. In some cases, generic methods such as 3DGS fail to reconstruct certain viewpoints reliably. These observations demonstrate that Thermal4D is effective not only for dynamic thermal reconstruction but also for static thermal scene modeling.

### 5.4. Quantitative Comparisons

#### 5.4.1. Camera Pose Registration Completeness

Reliable camera pose registration is essential for thermal scene reconstruction, especially in low-texture and low-contrast environments. We, therefore, compare our VGGT-based pose initialization pipeline with COLMAP, a widely used structure-from-motion framework. Both methods are applied to contrast-enhanced 8-bit thermal images extracted from MVTD.

As reported in Table 1, COLMAP frequently fails to register a sufficient number of views in dynamic thermal scenes because weak texture and unstable feature matching make the registration process difficult. In contrast, VGGT successfully registers all evaluated views for all three sequences. These results indicate that VGGT provides substantially better robustness and registration completeness than conventional SfM under thermal imaging conditions, thereby offering a more reliable initialization stage for subsequent dynamic reconstruction.

#### 5.4.2. Scene Reconstruction Performance on Dynamic Scenes

Table 2 reports the quantitative comparison on the dynamic sequences in the MVTD dataset. Overall, Thermal4D achieves the best performance across all three scenes and all three metrics. Compared with K-Planes and 4DGS, our method consistently obtains higher PSNR and SSIM values and lower LPIPS scores, indicating improvements in photometric fidelity, structural consistency, and perceptual quality. These gains are particularly evident in the Robotic Arm and Heating Platform sequences, where the motion patterns are more complex and the thermal structures are less texture-rich.

Compared with ENeRFi and Instant-NGP+T, which are adapted from general neural rendering pipelines within EasyVolcap, Thermal4D also shows clear advantages. This suggests that directly applying generic dynamic view synthesis methods to thermal imagery is insufficient to address the radiometric ambiguity and weak structural cues inherent in thermal data.

These results indicate that Thermal4D is particularly effective in scenes with articulated motion, thermal discontinuities, and weak texture, where generic rendering methods often struggle to produce reliable reconstructions.

#### 5.4.3. Efficiency Comparison on Dynamic Scenes

Thermal4D also achieves competitive efficiency on the MVTD dynamic benchmark when evaluated on a single NVIDIA RTX 3090 GPU. As shown in Table 3, although the proposed method introduces additional computation through TherHiLo and ATM, it remains close to 4DGS in both training time and rendering speed, while substantially outperforming K-Planes in overall runtime efficiency. Specifically, Thermal4D requires 47 min of training and renders at 29 FPS, compared with 38 min and 32 FPS for 4DGS, and 132 min and 0.3 FPS for K-Planes. The peak GPU memory usage of Thermal4D is higher than that of 4DGS because of the additional thermal feature extraction branch, but it remains within a practical single-GPU budget. These results indicate that the proposed method improves reconstruction quality with only moderate additional computational overhead.

#### 5.4.4. Scene Reconstruction Performance on Static Scenes

Table 4 reports the quantitative comparison on supplementary static scene benchmarks, including the UAV subset of TI-NSD and the NTR dataset. The TI-NSD UAV subset is used to assess performance under outdoor long-range thermal imaging conditions, while the NTR dataset provides an additional challenging benchmark for comparison with NTR-Gaussian. On the UAV subset of TI-NSD scenes, Thermal4D achieves the best overall performance across PSNR, SSIM, and LPIPS. Compared with the generic 3DGS baseline, the gains are substantial, showing that thermal-specific modeling is important even in the static setting. Thermal3D-GS improves over 3DGS by incorporating thermal imaging priors, while NTR-Gaussian further introduces physically meaningful thermal parameters. Nevertheless, Thermal4D still produces the strongest overall results on TI-NSD, indicating that the combination of frequency-aware representation learning and physics-aware rendering benefits both dynamic and static thermal reconstruction.

On the NTR dataset, NTR-Gaussian achieves the best result, while Thermal4D ranks second and remains competitive across all three metrics. This result is reasonable because NTR-Gaussian is explicitly optimized for thermodynamic parameter estimation in nighttime thermal reconstruction. Even so, the strong performance of Thermal4D on this scene demonstrates that the proposed framework generalizes well beyond the MVTD and TI-NSD settings.

### 5.5. Ablation Study

To evaluate the contribution of each component in Thermal4D, we conduct ablation studies on both MVTD and TI-NSD. The results are summarized in Table 5.

Removing TherHiLo leads to a clear performance drop on both datasets, especially in LPIPS, which indicates that frequency-aware thermal representation is important for preserving perceptual structure and local detail. Removing ATM also degrades the results, with a more noticeable impact on TI-NSD, where longer-range imaging and atmospheric effects are more relevant. This confirms the value of physics-aware radiometric modeling for thermal rendering.

When the DINO-based feature supervision term is removed, the performance decreases slightly, particularly in perceptual quality, suggesting that high-level feature guidance helps regularize reconstruction beyond pixel space. Finally, replacing the 14-bit thermal input in TherHiLo with 8-bit images also causes a consistent performance drop, especially on MVTD. This observation verifies that preserving the original radiometric precision of thermal measurements is beneficial for motion-sensitive feature extraction and dynamic reconstruction.

Taken together, these ablation results show that the gains of Thermal4D do not arise from a single component. Instead, the improvements come from the complementary effects of frequency-aware thermal attention, atmospheric transmission modeling, feature-level supervision, and high-precision thermal input.

## 6. Conclusions

In this paper, we presented Thermal4D, a physics-informed framework for dynamic scene reconstruction using only thermal infrared imagery. Built upon 3DGS, Thermal4D introduces a frequency-aware attention module (TherHiLo) and an atmospheric transmission module (ATM) to address the key challenges of thermal imaging, including low contrast, weak texture, and radiometric distortion. In addition, high-precision 14-bit thermal frames are incorporated into TherHiLo to provide richer radiometric cues for feature learning, while the ATM improves the physical consistency of thermal rendering by modeling emissivity and atmospheric transmission effects.

Experimental results on both dynamic and static thermal datasets demonstrate that Thermal4D consistently outperforms representative existing methods in terms of reconstruction quality and perceptual fidelity. The ablation studies further verify the effectiveness of the proposed components. These results suggest that Thermal4D is a promising solution for thermal scene reconstruction in challenging environments. Future work will extend the benchmark to more diverse and outdoor dynamic thermal scenes and explore model compression and acceleration techniques for real-time inference and efficient deployment on edge devices.

## Figures and Tables

**Table 1:** Comparison of camera pose registration completeness on dynamic thermal sequences using 8-bit inputs. The fractions denote the number of successfully registered views over the number of evaluated views. For the Robotic Arm and Heating Platform sequences, three viewpoints are excluded after preprocessing and data-quality screening, leaving 17 evaluated viewpoints for each sequence. VGGT denotes the pose initialization method used in our pipeline.

	COLMAP	VGGT
Little Sun	1/20	20/20
Robotic Arm	2/17	17/17
Heating Platform	2/17	17/17

**Table 2:** Quantitative comparison on dynamic thermal scenes from the MVTD dataset. The best and second-best results are indicated in bold and underlined, respectively.

Method	Little Sun			Robotic Arm			Heating Platform		
	PSNR↑	SSIM↑	LPIPS↓	PSNR↑	SSIM↑	LPIPS↓	PSNR↑	SSIM↑	LPIPS↓
K-plane	18.32	0.6743	0.5251	28.19	0.8784	0.4157	28.08	0.8337	0.5348
ENeRFi	18.21	0.6538	0.5302	27.55	0.8439	0.4481	27.19	0.8125	0.5441
Instant-NGP+T	18.19	0.6525	0.5298	27.31	0.8416	0.4524	27.13	0.8129	0.5481
4DGS	19.13	0.6968	0.5025	30.67	0.9055	0.4025	29.50	0.8413	0.5254
**Thermal4D (ours)**	**22.47**	**0.7323**	**0.4753**	**32.31**	**0.9342**	**0.3723**	**31.73**	**0.8624**	**0.5089**

**Note:** The proposed method is highlighted in bold in the Method column. Bold numerical values indicate the best performance in each scene, underlined numerical values indicate the second-best performance, ↑ indicates that higher values are better, and ↓ indicates that lower values are better.

**Table 3:** Efficiency comparison on the MVTD dynamic reconstruction benchmark. We report total training time, rendering speed (FPS), and peak GPU memory usage under the same hardware setting.

Method	Training Time (min)	FPS	Peak GPU Memory (GB)
K-Planes	132	0.3	2.33
4DGS	38	32	7.23
Thermal4D (ours)	47	29	8.78

**Table 4:** Quantitative comparison on static thermal scenes from TI-NSD and NTR dataset.

Scene	Method	PSNR↑	SSIM↑	LPIPS↓
UAV1	3DGS	32.29	0.9218	0.154
	Thermal3D-GS	36.34	0.9552	0.1195
	NTR-Gaussian	34.62	0.9376	0.1512
	4DGS	35.88	0.9458	0.1487
	**Thermal4D (ours)**	**37.75**	**0.9638**	**0.1079**
UAV2	3DGS	34.47	0.9618	0.0880
	Thermal3D-GS	36.87	0.9664	0.0882
	NTR-Gaussian	35.59	0.966	0.0912
	4DGS	36.17	0.9566	0.1008
	**Thermal4D (ours)**	**38.24**	**0.9724**	**0.0821**
UAV3	3DGS	35.96	0.9657	0.0804
	Thermal3D-GS	36.40	0.9655	0.0821
	NTR-Gaussian	36.27	0.9659	0.0831
	4DGS	36.19	0.9622	0.0905
	**Thermal4D (ours)**	**37.52**	**0.9674**	**0.0784**
UAV4	3DGS	33.92	0.9559	0.1597
	Thermal3D-GS	36.73	0.9602	0.1577
	NTR-Gaussian	35.24	0.9535	0.1639
	4DGS	36.44	0.9546	0.1827
	**Thermal4D (ours)**	**37.96**	**0.9659**	**0.1328**
UAV5	3DGS	35.72	0.9586	0.1406
	Thermal3D-GS	37.02	0.9611	0.1404
	NTR-Gaussian	36.71	0.9597	0.1502
	4DGS	36.25	0.953	0.1695
	**Thermal4D (ours)**	**37.88**	**0.9665**	**0.1209**
UAV6	3DGS	36.84	0.9645	0.0776
	Thermal3D-GS	36.92	0.9648	0.0793
	NTR-Gaussian	36.54	0.9649	0.0793
	4DGS	36.63	0.9606	0.091
	**Thermal4D (ours)**	**38.02**	**0.9673**	**0.0742**
NTR Dataset	3DGS	31.29	0.955	0.1643
	Thermal3D-GS	30.61	0.9603	0.1576
	NTR-Gaussian	**31.43**	**0.964**	**0.1501**
	4DGS	29.17	0.9618	0.1641
	**Thermal4D (ours)**	31.37	0.9627	0.1548

**Note:** The proposed method is highlighted in bold in the Method column. Bold numerical values indicate the best performance in each scene, underlined numerical values indicate the second-best performance, **↑** indicates that higher values are better, and **↓** indicates that lower values are better.

**Table 5:** Ablation study on MVTD and TI-NSD. We evaluate the effects of the TherHiLo module, the atmospheric transmission module (ATM), the feature-level supervision term, and the use of 14-bit thermal input.

Method	MVTD			TI-NSD		
	PSNR↑	SSIM↑	LPIPS↓	PSNR↑	SSIM↑	LPIPS↓
Thermal4D w/o TherHiLo	26.72	0.8062	0.4733	36.44	0.9612	0.1152
Thermal4D w/o ATM	28.17	0.8291	0.4623	36.29	0.9573	0.1239
$\mathrm{Thermal}4D\;w/o\;L_{feat}$	27.67	0.8226	0.4687	36.80	0.9629	0.1032
TherHiLo w/o 14-bit input	27.01	0.8137	0.4664	36.52	0.9610	0.1086
**Thermal4D (ours)**	**28.84**	**0.843**	**0.4522**	**37.90**	**0.9672**	**0.0994**

**Note:** The proposed method is highlighted in bold in the Method column. Bold numerical values indicate the best performance in each scene, **↑** indicates that higher values are better, and **↓** indicates that lower values are better.

## Data Availability

The MVTD dataset and related data used in this study are not posted in a public repository at this stage due to institutional data-management and transfer restrictions following the authors’ affiliation transition. The data may be obtained from the corresponding author upon reasonable request and subject to approval of the relevant institutional requirements.

## Abbreviations

The following abbreviations are used in this manuscript:

AGC	Automatic Gain Control
ATM	Atmospheric Transmission Module
COLMAP	Structure-from-Motion and Multi-View Stereo Pipeline
DINOv2	Self-Distilled Vision Transformer v2
DTM	Deformable Token Merging
LPIPS	Learned Perceptual Image Patch Similarity
LWIR	Long-Wave Infrared
MLP	Multilayer Perceptron
MVTD	Multi-View Thermal Dynamic Dataset
PSNR	Peak Signal-to-Noise Ratio
SSIM	Structural Similarity Index Measure
SfM	Structure from Motion
ViT	Vision Transformer
TherHiLo	Thermal High–Low Attention
